# Protection against Amoebic Liver Abscess in Hamster by Intramuscular Immunization with an *Autographa californica* Baculovirus Driving the Expression of the Gal-Lectin LC3 Fragment

**DOI:** 10.1155/2015/760598

**Published:** 2015-05-19

**Authors:** Dulce María Meneses-Ruiz, Hugo Aguilar-Diaz, Raúl José Bobes, Alicia Sampieri, Luis Vaca, Juan Pedro Laclette, Julio César Carrero

**Affiliations:** ^1^Departamento de Inmunología, Instituto de Investigaciones Biomédicas, Universidad Nacional Autónoma de México, Ciudad Universitaria, 04510 México, DF, Mexico; ^2^Departamento de Biología Celular, Instituto de Fisiología Celular, Universidad Nacional Autónoma de México, Ciudad Universitaria, 04510 México, DF, Mexico

## Abstract

In a previous study, we demonstrated that oral immunization using* Autographa californica* baculovirus driving the expression of the Gal-lectin LC3 fragment (AcNPV-LC3) of* Entamoeba histolytica* conferred protection against ALA development in hamsters. In this study, we determined the ability of AcNPV-LC3 to protect against ALA by the intramuscular route as well as the liver immune response associated with protection. Results showed that 55% of hamsters IM immunized with AcNPV-LC3 showed sterile protection against ALA, whereas other 20% showed reduction in the size and extent of abscesses, resulting in some protection in 75% of animals compared to the sham control group. Levels of protection showed a linear correlation with the development and intensity of specific antiamoeba cellular and humoral responses, evaluated in serum and spleen of hamsters, respectively. Evaluation of the Th1/Th2 cytokine patterns expressed in the liver of hamsters showed that sterile protection was associated with the production of high levels of IFN*γ* and IL-4. These results suggest that the baculovirus system is equally efficient by the intramuscular as well as the oral routes for ALA protection and that the Gal-lectin LC3 fragment is a highly protective antigen against hepatic amoebiasis through the local induction of IFN*γ* and IL-4.

## 1. Introduction

*Entamoeba histolytica* is the protozoan parasite that causes amoebiasis in humans. This disease is widely prevalent in population of developing countries with poor living conditions and hygiene. The parasite has been estimated to infect 40 million people around the world, although the real number of* E. histolytica* cases is unknown due to the inclusion in this estimation of cases with the morphologically identical* E. dispar* and* E. moshkovskii* species. However, since* E. histolytica* is the unique specie considered as pathogen for humans, it appears to be responsible for 10 million cases of amoebic dysentery/amoebic liver abscesses and about 100,000 deaths every year [1]. In Mexico, amoebiasis was ranked as the sixth highest cause of morbidity with an incidence of 498 cases per 100,000 habitants in 2008 [2].

Amoebiasis treatment relies on the use of imidazole derivatives such as metronidazole, which is highly effective but has the drawback of inducing side effects, is mutagenic at high concentrations, and induces the development of cellular resistance [3]. Thus, there are reports of* in vitro* induction of resistant cultures to high concentrations of metronidazole by continuous exposure to increasing concentrations of the drug as well as the description of patients with amoebic liver abscesses reluctant to the treatment [4]. Another option that has been shuffled for controlling amoebiasis is the development of a vaccine. In this regard, there have been many trials of immunization in experimental animals using different amoeba antigens in combination with adjuvant [5].

The galactose-binding lectin is among the antigens most commonly used for protection assays. This is a protein complex of three subunits that are preferably located at the surface of the parasite and whose main component, the heavy subunit of 170 kDa, is also one of the most immunogenic* E. histolytica* molecules [6]. Along with other proteins, such as the family of serine-rich proteins [7] and the 29 kDa cysteine-rich Alkyl hydroperoxide reductase [8], the Gal-lectin is considered as one of the main targets for an effective vaccine against amoebiasis. The gal-lectin, with its cysteine-rich portion of the 170 kDa lectin subunit, is the target for serum of 95% of patients with amoebic liver abscess [9] as well as IgG and IgA anti-Gal-lectin antibodies recovered from serum and feces of patients with intestinal amoebiasis, respectively [10, 11]. Oral or nasal immunization of mice, gerbils, and nonhuman primates with the cystein-rich section of galactose-inhibitable lectin LC3 and cholera toxin as adjuvant induced high level of specific serum IgG and fecal IgA [12, 13] antibodies that inhibit* in vitro E. histolytica* adherence to CHO cells [14]. Moreover, intraperitoneal immunization of gerbils with the LC3 fragment with Titermax adjuvant elicited IgG antibodies that conferred 71% of protection against ALA [15]. Recently, it was demonstrated that LC3 is one of the main targets of antibodies elicited by natural infection of female baboons with* E. histolytica* [16]. Thus, 73% and 46% of such animals showed serum anti-LC3 IgG and IgA antibodies, respectively, and 49% exhibited fecal anti-LC3 secretory IgA antibodies. Noteworthy, the specificity of recognition of epitopes in LC3 and the native Gal-lectin by the infected baboons was similar to the specificity of recognition of human asymptomatic subjects and ALA patients [16].

Although promising results have been obtained in protection assays against amoebiasis using various experimental models such as mice, hamsters, and gerbils, the use of these strategies to protect humans in the future is hampered by the use of adjuvants that are potentially toxic and proinflammatory to mammals, such as bacterial toxins or oil-based adjuvants. In a previous report, we proposed the use of viral vectors such as the baculovirus as a strategy for the delivery of amoebic antigens in studies of protection [17]. Baculoviruses are insect viruses capable of infecting mammalian cells, but not of replicating in them. The most promising is* Autographa californica*, an envelope and double-stranded DNA nucleopolyhedrovirus that can drive the expression of foreign genes in mammalian cells without causing cytotoxic effects [18]. This virus has the ability to transduce a wide range of mammalian cells, including liver and kidney cells, and therefore it has been proposed as a delivery vector for human gene therapy, mainly as oncolytic agent. In this sense,* A. californica* has been proposed as a tool for targeting and transferring therapeutic genes into carcinoma cells from patients with prostate [19], colorectal [20], and lung [21] cancer as well as ameliorating the collateral ischaemia in organ transplantation [22]. Noteworthy, from 27 nonhuman virus species that are in preclinical studies,* A. californica* was classified in the category of “negligible” regarding the relative environmental risk [18], suggesting that its use in human for any medical purpose, including gene therapy and vaccines, is safe. As vaccine delivery strategy, the baculovirus* A. californica* has the advantage of priming the immune response by itself, innate and adaptive as well as humoral and cellular [23], preventing the need for adjuvants with potential adverse effects such as bacterial toxins and oil-based formula. Thus, baculovirus displaying target antigens on their surface and/or driving the expression of them under the control of a cytomegalovirus promoter has been highly efficient in inducing immune responses that confer protection against viral and parasitic infections in experimental models [24–35].

In a previous report, we described the potential of an* A. californica* nucleopolyhedrovirus (AcNPV) system driving the expression of* E. histolytica* Gal-lectin LC3 fragment of conferring sterile or partial protection against ALA in 79% of orally and 21% of nasally immunized hamsters. Although the protection was associated with the development of an antiamoebic cellular immune response measured in spleen, the underlying cytokines and cell populations responsible were not identified. Herein, we complement the previous study by evaluating the potential of an intramuscular immunization with the AcNPV/CMV/Gal-lectin LC3 system for conferring protection against ALA in hamsters, determining whether this route of immunization confer a greater level protection than the mucosal delivery and evaluating in the liver the locally produced cytokines and cell populations responsible for protection.

## 2. Materials and Methods

### 2.1. Cells and Cultures

Axenic HM1: IMSS trophozoites were maintained in TYI-S-33 medium supplemented with 15% of adult bovine serum (Biofluids International Inc., MD, USA) and 3% of Diamond's vitamin mix (JRH Biosciences, Kansas, USA). Trophozoites virulence was maintained through successive passages into hamster's liver.

sf9 insect cells (*Spodoptera frugiperda*) (Invitrogen, San Diego, CA) were cultivated as a suspension in Grace's medium (Invitrogen, USA) supplemented with 10% heat inactivated fetal bovine serum (Invitrogen, Carlsbad, CA), 1% penicillin/streptomycin (100 U/mL), and 0.1% pluronic F-68 (Invitrogen, USA). AcNPV-LC3 baculovirus production was described in [17]. Recombinant AcNPV-LC3 baculovirus was amplified by infecting sf9 insect cells at a multiplicity of infection (MOI) of 0.1 and purifying them from culture's supernatants 6 days after and concentrated by ultracentrifugation, resuspended in PBS and titered by using a plaque assay following the manufacturer's instructions (Invitrogen, USA).

### 2.2. Immunization and Challenge Protocols

Male Syrian golden hamsters (*Mesocricetus auratus*) 4 to 6 weeks of age were maintained free of pathogens with water and food* ad libitum*. Following a protocol approved by the Institutional Animal Care Committee, animals were divided in 2 groups of 20 hamsters (AcNPV-WT and AcNPV-LC3 groups) and 1 group of 10 hamsters (sham group). Hamsters of groups AcNPV-WT and AcNPV-LC3 were intramuscularly immunized with three doses at two-week intervals with 1 × 10^8^ PFU of wild-type or LC3 recombinant baculovirus prepared in 20 *μ*L PBS, pH 7.4, respectively. Sham group received 20 *μ*L of PBS by intramuscular route following the same protocol. Two weeks after the last immunization, all animal were infected by intraportal route with virulent* E. histolytica* trophozoites. In brief, hamsters were anesthetized with sodium pentobarbital (50 mg/kg; Anestesal, Pfizer), a laparatomy practiced in aseptic conditions, and 10^6^ trophozoites in 100 *μ*L PBS were directly injected in the portal vein. The site of injection was immediately obtruded by applying a gel foam pad, intestine carefully returned to the abdominal cavity, and the abdominal layers sutured with surgical staples (Reflex 9, USA). Blood samples were collected from all animals before treatment and after immunization prior to challenge. Sera were obtained by centrifugation and stored at −70°C until use.

### 2.3. ELISA

96 well plates were coated overnight with 500 ng/well of trophozoite's total extract in carbonate buffer, pH 9.6 at 4°C. After blocking with 1% BSA-Tween 20, hamster's sera diluted in 1% BSA (1 : 50) were added to the wells and incubated 1 h at 37°C. After extensive washings with PBS-Tween 20, a HRP-conjugated anti-hamster IgG antibody (Becton Dickinson, USA) was added to 1 : 1000 dilution and incubated for 1 h at 37°C. The wells were washed and the antigen-antibody complexes were developed with OPD and read at 490 nm in a spectrophotometer.

### 2.4. Cytokine Determination

Cytokine determination was performed in soluble liver extract from hamsters with commercially available mouse IFN*γ*, IL-12 p70, IL-4, and IL-10 ELISA MAX Standard kits (BioLegend) according to manufacturer's instruction (capture ELISA). Each cytokine assay was performed in triplicate each time. In brief, liver extracts were obtained by sonication (three times, 30 sec each on ice) of tissue fragments, containing or not ALA, in the diluent solution provided with the kit in the presence of complete protease inhibitor cocktail (Roche). Extracts were centrifuged at 5000 rpm for 10 min at 4°C and the soluble fraction, once determining protein concentration by the Lowry method, stored at −20°C until use. For cytokine determination, each well was added with 5 *μ*g of extract in 100 *μ*L of diluent solution and incubated for 2 h at room temperature. Secondary antibodies were used 1 : 200 dilution in diluent solution for 1 h at room temperature and the immune complex developed using Avidine-HRP/TMB substrate and reading at 650 nm in a spectrophotometer.

### 2.5. Spleen Cells Proliferation by Flow Cytometry

After sacrifice at 7 days after challenge, hamster's spleens were removed and its cellular fraction obtained by perfusion in 2 mL supplemented RPMI medium. The cells were incubated with hemolysis solution and washed by centrifugation with RPMI media. After the erythrocytes lysis, 1 × 10^6^ lymphocytes were gating with 1 mL of PBS/0.1% BSA solution and incubated with 5 mM/mL of CFSE (CellTrace CFSE Cell Proliferation Kit C34554, INVITROGEN) during 10 min at 37°C. After the incubation, the cells were washed in RPMI media and cultivated in 96-well plates at 37°C with 5% CO_2_ and 95% humidity for 72 h in RPMI medium alone or stimulated with concanavalin A (1 *μ*g/well), or amebic total extract (50 *μ*g/well). Finally, the lymphocytes were harvested and fixed with 3.7% of formaldehyde solution. All the treatments were carried out by triplicate for each animal. Samples were analyzed on a FACSCanto II flow cytometer (BD) on a minimum of 20,000 events using the DIVA software. The computational analysis was carried out in FACSDiva Software Version 6. The statistical analysis was performed with Batch Analysis Report. Proliferation data from each experimental group was obtained by pooling the means of the triplicate for each animal and is presented as mean ± SD.

### 2.6. Assay for Liver Function

Blood samples were obtained from hamsters at day of sacrifice. Serum was separated from the blood samples by centrifugation at 5000 rpm for 10 min at room temperature. The serum marker of liver function alanine amino transferase (ALT) was determined by the method EAGLE-UV at Department of Pathology, Faculty of Veterinary Medicine, UNAM.

### 2.7. Statistical Analysis

The comparison of the infection rates was done using Fisher's exact test. The Kruskal-Wallis test was used to compare antibody and proliferation of lymphocytes between groups. A *P* value < 0.05 was considered statistically significant in these analyses. All statistical analyses were performed using SPSS statistical software version 19.0.

## 3. Results

### 3.1. Intramuscular Immunization with AcNPV-LC3 Inhibits the Development of ALA in Hamsters

In order to evaluate the potential of recombinant baculovirus driving the expression of the amoebic Gal-lectin LC3 fragment of protecting against ALA, hamsters were intramuscularly immunized with the viral particles and intraportally challenged with virulent* E. histolytica* trophozoites. Description on how the recombinant baculoviruses were obtained and evaluated for driving the expression of the Gal-lectin LC3 fragment was previously reported [17].

**Figure 1:**
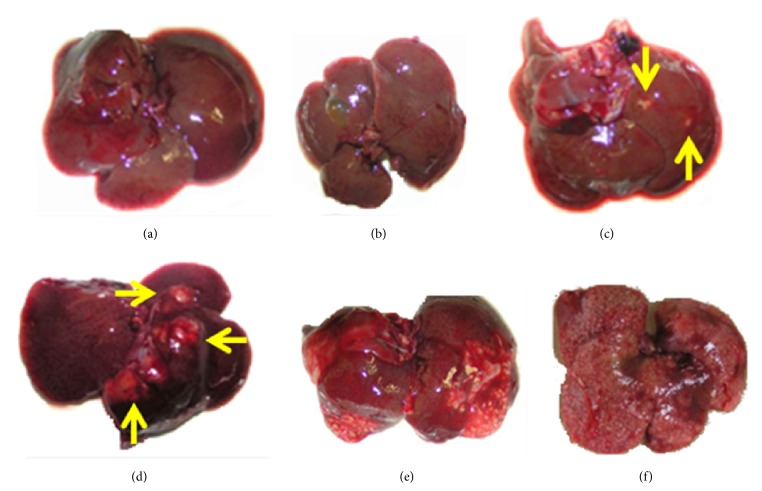
Macroscopical evaluation of protection from ALA challenge in hamsters intramuscularly immunized with Ac-NPV-LC3. Hamsters were IM immunized with baculovirus or PBS and then challenged with virulent* E. histolytica* trophozoites by intraportal route. After sacrifice, livers were exscinded showing different grades of ALA. Sterile protection ((a) and (b)) and partial protection showing few abscesses (c) or no more than 5 large abscesses usually located in one lobe (d) (yellow arrows) were observed in AcNPV-LC3 and AcNPV-WT immunized animals. Massive abscesses development ((e) and (f)) was mainly observed in hamsters nonimmunized but infected.

A comparative macroscopic description of livers showed variable grades of ALA development (Figure 1). Massive development of ALA extended throughout the liver was observed in 8 out of 10 (80%) nonimmunized but challenged hamsters (Sham group; Figures 1(e) and 1(f) and Table 1). By contrast, the development of ALA was variable in immunized and challenged animals. Thus, 11 out of 20 hamsters immunized with the recombinant baculovirus AcNPV-LC3 and 5 out of 20 immunized with the wild-type baculovirus showed no gross (Figures 1(a) and 1(b)) or microscopic evidence (not shown), of ALA development, suggesting that intramuscular immunization with the baculovirus* per se* is capable of giving up to 25% sterile protection, which is increased to more than twice (55%) by immunizing with the recombinant baculovirus of LC3 (Table 1). Moreover, within the immunized animals that developed ALA, 1 out of 5 animals immunized with the wild virus and 4 out of 9 immunized with AcNPV-LC3 showed partial protection (Table 1), some showing few millimetric abscesses scattered throughout the liver (Figure 1(c)) or no more than 5 large abscesses usually located in one lobe (Figure 1(d)).

The protection against the development of ALA was confirmed by analyzing the hepatomegaly associated and by testing liver function, in particular the determination of alanine aminotransferase (ALT), a pyridoxal cytoplasmic phosphate-dependent enzyme involved in cellular nitrogen metabolism, amino metabolism acid, and liver gluconeogenesis. ALT levels are often low in blood but increased in the case of liver diseases or events that include damage of that tissue [36]. The results showed high levels of ALT, about three times above the reference value, in the serum of nonimmunized and infected animals (sham group), which correlates with an increase of more than twice the weight of the liver as a result of abscesses (Table 1). By contrast, the liver weight and ALT levels in the serum of immunized animals correlated with the degree of protection, being these normal values in hamsters fully protected (ALT < 87 U/L and liver around 6 g) and intermediate in animals showing partial protection (Table 1).

Overall, it can be considered that 75% of hamsters immunized with the recombinant baculovirus (*P* < 0.002 versus WT group by Fisher's test) and 30% of hamsters immunized with the wild baculovirus (*P* < 0.02 versus AcNPV-LC3 group by Fisher's test) showed any evidence of protection against the development of ALA (Table 2).

### 3.2. Intramuscular Immunization with the Recombinant Baculovirus AcNPV-LC3 Induces High Levels of Serum Anti-Amoebic Antibodies in Hamsters

**Figure 2:**
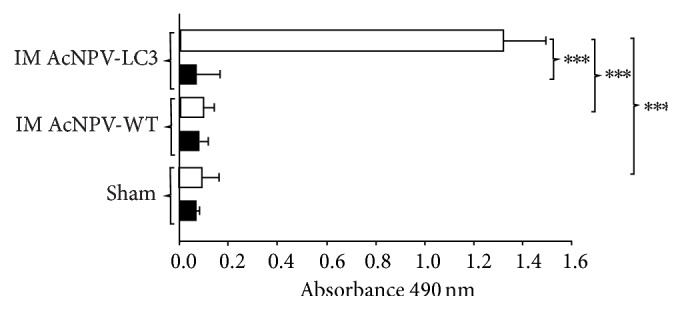
Humoral anti-*E. histolytica* immune responses in AcNPV-LC3 intramuscular immunized and challenged hamsters. IgG antibodies in sera against total extract of* E. histolytica* trophozoites were measured by ELISA at day 0 (preimmune sera, black bars) and day 35, once finalizing the immunization protocol (postimmune sera, 7 days prior to intraportal challenge with trophozoites; white bars). Bars represent mean optical densities (^*∗∗∗*^ *P* < 0.001).

Analysis of sera from hamsters by ELISA against whole amoeba antigen showed the development of high levels of specific IgG antibodies after intramuscular immunization with the recombinant baculoviruses AcNPV-LC3 (Figure 2; white bars; *P* < 0.001 versus control groups). Since the sera of animals immunized with wild baculovirus, as well as sham control animals, did not show reaction against the antigen of amoeba, antibodies from hyperimmune sera of immunized hamsters were assumed to be directed against the LC3 fragment Gal-lectin (Figure 2).

### 3.3. Intramuscular Immunization with the Recombinant Baculovirus AcNPV-LC3 Induces a Cell-Mediated Antiamoebic Response in Hamsters

**Figure 3:**
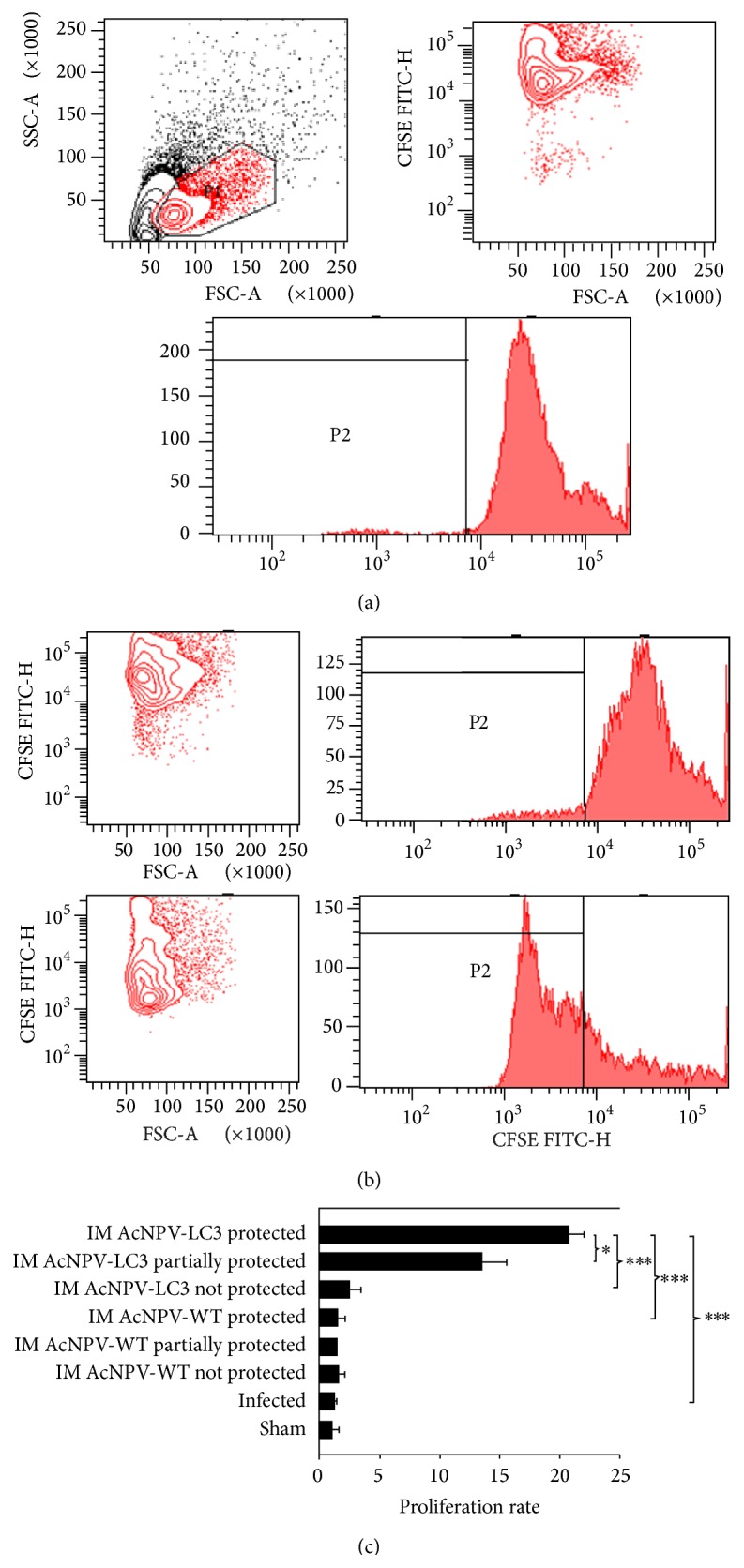
Cellular anti-*E. histolytica* responses in AcNPV-LC3 intramuscular immunized and challenged hamsters. Cellular immune response was evaluated in splenocytes after immunization and challenge (day 49) by staining of the harvested cells with CFSE and stimulation with* E. histolytica* total extract during 72 h followed by flow cytometry analysis. In (a), the first panel shows dot plot of splenocytes stained with CFSE without antigenic stimulation. The cell population selected according to their size and granularity (P1 region) and its corresponding dot plot and histogram are shown (a). (b) shows a representative dot plot and histogram of IM AcNPC-LC3 splenocytes from nonprotected (upper) and sterile protected (bottom) hamsters. (c) shows the proliferation rate of all experimental groups classified by protection level. Bars represent mean of percentage of proliferation (^*∗*^ *P* < 0.05; ^*∗∗∗*^ *P* < 0.001).

Antigen specific proliferation of lymphocytes was determined in sensitized spleen cells from hamsters, by incubating splenocytes in the presence of whole amoeba antigen. The results showed that the level of proliferation correlated with the degree of protection obtained with the recombinant baculovirus AcNPV-LC3 (Figure 3), being greater in the immunized animals protected entirely (rate 20) followed by those which were partially protected (rate 13; Figure 3(c), *P* < 0.05 between them; *P* < 0.001 versus the other groups). Proliferation in the AcNPV-LC3 immunized but not protected hamsters was twice of the animals from control groups, but not statistically different. By contrast, animals not protected and even those totally or partially protected by immunization with the wild baculovirus did not show any degree of splenocyte's proliferation against whole amoeba antigen (Figure 3).

### 3.4. Protection against ALA Correlated with the Local Production of IFN*γ* and IL-4

**Figure 4:**
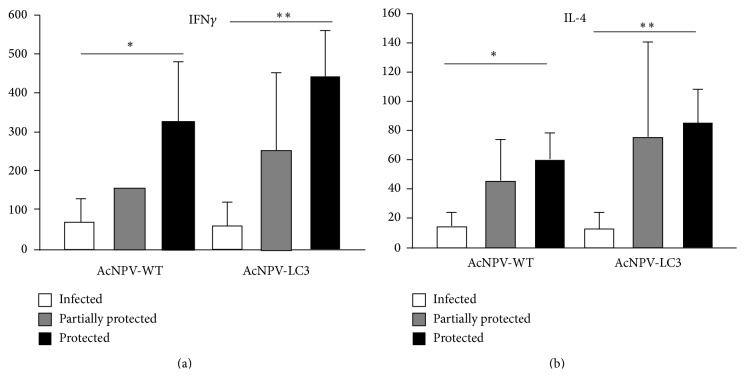
IFN*γ* and IL-4 local productions in liver parenchyma of hamsters intramuscularly immunized with AcNPV-WT, AcNPV-LC3 and challenged. Cytokines in soluble livers extracts from hamsters of AcMPV-WT and AcMPV-LC3 groups were evaluated by ELISA. Concentrations in extracts from immunized and nonprotected hamsters (white bars), partially protected hamsters (grey bars), and hamsters with sterile protection (black bars) are shown. The results show a direct relationship between IL4 (a) and IFN*γ* (b) expression and protection degree (^*∗*^ *P* < 0.05, ^*∗∗*^ *P* < 0.01).

In order to identify soluble elements of the local cellular immune response associated with the protection obtained against ALA, the Th1 (IFN*γ* and IL-12) and Th2 (IL-4 and IL-10) associated cytokines in extracts of livers from protected hamsters were evaluated. The results animals immunized with AcNPV-LC3 and completely protected had on average 9-fold more IFN*γ* than infected animals (450 pg/mL versus 50 pg/mL, *P* < 0.01), and almost twice the levels in partially protected hamsters, but with no statistical significance (Figure 4(a)). Similarly, there was no statistical difference between the IFN*γ* levels from liver of the partially protected and the nonprotected animals. In the same way, this trend was observed in AcNPV-WT group, where totally protected animals have 6 times average of IFN*γ* more than infected animals (396.5 pg/mL versus 65.2 pg/mL, *P* < 0.05, Figure 4(a)) and twice with respect to animals that showed partial protection. Finally, no difference between groups was observed for the other Th1 cytokine IL-12 (data not shown).

With respect to the Th2 cytokine profile, differences were only observed in the levels of IL-4, but not IL-10 (data not shown). As with IFN*γ*, IL-4 was on average 8-fold higher in the livers of animals fully protected compared with immunized and infected animals (80 pg/mL versus 10 pg/mL, *P* < 0.01, Figure 4(b)). In the same way, the partially protected hamsters showed a tendency to have higher concentrations of IL-4 than the infected, but the difference was not statistically significant. With regard to AcNPV-WT immunized group, the totally protected animals showed IL-4 six times higher more than infected animals.

## 4. Discussion

In a previous study, we demonstrated the potential of* A. californica* nucleopolyhedrovirus (AcNPV) driving the expression of the* E. histolytica* Gal-lectin LC3 fragment of conferring sterile or partial protection against ALA in 79% of orally and 21% of nasally immunized hamsters, but the underlying cytokines and cell populations responsible were not identified [17]. In this report we aimed to determine whether the intramuscular immunization of hamsters with the same AcNPV-LC3 recombinant baculovirus was able to confer a greater level of protection than the mucosal vaccination against the development of ALA following an intraportal challenge with virulent amoebic trophozoites and whether the protection was correlated with a particular type of local immune response.

The results showed that intramuscular immunization of hamsters with the AcNPV-LC3 recombinant baculovirus confers an overall level of protection similar to that previously obtained with the oral route (75% versus 79%, resp.). Moreover, the sterile protection levels obtained by both routes were also similar (55% oral versus 58% intramuscular). This result was unexpected because being ALA a systemic type infection, it was more probably that the intramuscular route of immunization had higher efficiency to induce a systemic protective response than the oral route. Therefore, this supports the proposal by our group and others that the oral route may be the appropriate to administer a vaccine against amoebiasis in general, because, in addition to the classic advantages of this route as its manageability, noninvasion, and no needs for specialized personnel, oral route triggers local and systemic immune responses that in addition to conferring protection against intestinal amoebiasis, may also protect against the development of liver abscesses if trophozoites manage to pass from the intestine to the liver. This is noteworthy as extraintestinal amoebiasis, and in particular, ALA is considered the main cause of human death by* E. histolytica* [37].

The effectiveness of the Gal-lectin as vaccine against amoebiasis has been extensively studied [5]. Recently, a new model of intestinal infection with* E. histolytica* in baboons (Papio sp) and the efficacy of nasal vaccination against amoebic colitis using a Gal-lectin synthetic peptide was reported [38]. Notably, a vaccination schedule of four doses of 1600 *μ*g peptide/nostril in combination with cholera toxin as adjuvant once a week was able to eradicate amoebic infection in all baboons by the 51st day after intracecal challenge with trophozoites. Moreover, serum IgG and IgA antibodies induced by natural infection of baboons with* E. histolytica* are directed to epitopes present in the LC3 fragment of the Gal-lectin, and this immunodominance is shared with humans carrying asymptomatic* E. histolytica* infection or recently cured of ALA [14]. These results, together with the obtained by our group, previously and reported here, using recombinant baculoviruses driving the expression of the LC3 fragment [17] as well as other protection assays using LC3 as immunogen [15], highly support that Gal-lectin, in particular its LC3 fragment, is the target of protective antibodies and cellular immune responses resolving intra- and extraintestinal amoebiasis, and, therefore, it should be the basic component of an effective vaccine against human amoebiasis.

The immune elements responsible for protection against intestinal amebiasis or ALA are not entirely known. In this study, we found an association of sterile protection against ALA with the local production of high levels of IFN*γ*, a cytokine of Th1 profile. This result is in agreement with previous studies where protection against ALA in hamsters correlated with the production of the same cytokine in protective assays using the EhCPADH surface complex as immunogen [39]. In addition, relevance of IFN*γ* as playing a role in the innate immunity against ALA came from studies in SCID mice with targeted disruption of the IFN*γ* receptor gene [40]. Moreover, T cell derived IFN*γ* and IL-17 were also essential for protection against intestinal amoebiasis in a murine model vaccinated with recombinant LecA fragment of the Gal-lectin, determined by adoptive T cell transfer and IFN*γ*/Il-17 neutralizing assays [41, 42]. The source of the protective IFN*γ* in our assays is unknown, but it could be from T cells responding specifically to the amoeba antigen as observed in the cell proliferation assays, in which the best protected animals showed higher response to the amoeba extract. However, NKTs cells as source of the early production of IFN*γ* in the liver during the ALA are also well known [43]. In this regard, interestingly, the wild-type baculovirus was able to confer up to 25% protection by itself, which was also associated with local production of high levels of IFN*γ*, suggesting that activation of the innate immune response by the baculovirus, probably IFN*γ* from local NKT cells, is partly responsible for the observed protection. Thus, it has been demonstrated that baculovirus recognition by TL9 leads to the production of various cytokines that triggers innate immunity, including IFN*γ* [23], which in turn has the potential to confer high levels of protection in several experimental models, such as influenza where intranasal immunization with a wild-type baculovirus protected 100% of mice from a lethal challenge [44].

In this report, a correlation of partial and sterile protection against ALA with the local production of high levels of IL-4 was also observed (Figure 4(b)). Although the production of IL-4 has been associated with susceptibility to intestinal infection by amoeba [45], a previous study by our group using lactoferrin as an oral therapy for the treatment of intestinal amebiasis in C3H/HeJ mice showed production of this cytokine in tissue of ceca from animal cured [46]. As we suggest in such paper, it is likely that the production of IL-4 is the result of a feedback mechanism for controlling excessive inflammation (a typical feature of the intestinal mouse and liver hamster amoebiasis), restoring the physiological anti-inflammatory state that predominates in both intestine and liver. IL-4 also augments fibroblast growth and collagen production during granulomas formation, a characteristic mark of the chronic development of ALA [47], a reaction trying to contain the infection. Thus, it is possible that the role of IL-4 depends on whether liver abscesses develop or not, so contributing to the formation of granulomas at later stages if ALA develops or decreasing excessive inflammation at earlier stages.

Protection against ALA in hamsters was also associated with the induction of serum anti-amoebic antibodies, particularly IgG (Figure 2). This is consistent with a variety of previous studies demonstrating that antibodies are important in protection against amoeba, particularly secretory IgA against intestinal amebiasis, and serum IgG and IgA against hepatic amoebiasis [48, 49]. It is possible that the high levels of IFN*γ* observed in protected hamsters have also contributed to the increased production of IgG antibodies mentioned, mainly IgG2a isotype (Th1 response), linking the two arms of the immune response in the liver infection by* E. histolytica*. Taken together, the results suggest that both responses, cellular and humoral anti-LC3 fragment of the Gal-lectin, are important for the development of resistance in hamsters against ALA.

Immunization in humans has always faced with the difficulty of identifying safe adjuvants that do not generate unwanted side effects. Most protection assays against intestinal and/or liver amoebiasis described above used toxins derived from bacteria or mineral or vegetable oils suspensions, which although inducing a good immune response also usually generate local or systemic inflammatory responses that can have implications for the health of the vaccinated subject [50]. In this work, we reinforce the probability of using recombinant baculovirus system as an alternative approach to the use of toxic adjuvants for the safe release of amoeba antigens into mammals, due to its* per se* capacity of stimulating the innate immune response, and, thus, conditioning the generation of a specific antiamoeba adaptive immune response. In this sense, many studies demonstrating the phenotypic and functional maturation of dendritic cells and macrophages as well as direct activation of B cells induced by AcNPV, resulting in antigen presentation and Th1 responses, have been reported [51, 52]. Noteworthy, the acceptability for using baculovirus in humans has been recently increased with ongoing trials of gene therapy based on the use of such viruses as delivery system [53], which lays the foundation for safe and extended use of recombinant baculovirus systems in other areas such as vaccines [54].

Finally, further studies of T cell adoptive transfer and neutralization of cytokines, IFN*γ* and probably IL-17, are needed for determining the mechanisms underlying protection against ALA in baculovirus-immunized hamsters, as well as immunization studies using AcNPV-LC3 in an animal model closer to humans such as primates, in order to determine the safety and effectiveness of this system with view to its use as a strategy of vaccination against amoebiasis in humans.

## Figures and Tables

**Table 1:** ALT levels in hamsters immunized with AcNPV-LC3 and challenged with *E. histolytica*.

Parameter	Infected	IM AcNPV-LC3 Total protection	IM AcNPV-LC3 Partially protection
Weight (g)	15 ± 3.9	6 ± 2.3	6.3 ± 3.2
ALT (U/L) <87	258.8 ± 163.2	32.2 ± 10.1	169.4 ± 112.3

**Table 2:** Infection rate in hamsters intramuscularly immunized with AcNPV-LC3 and intraportally infected with virulent *E. histolytica* trophozoites.

Groups	Infection rate (Infected/Total) %	Total protection %	Partially protection %	Protection efficacy %	*P* value (Fisher's Test)

Infected	8/10 (80%)	0	0	0	0
IM AcNPV-WT	15/20 (75%)	25	5	30	0.02 (vs IM AcNPV-LC3)
IM AcNPV-LC3	9/20 (45%)	55	20	75	0.002 (vs IM sham)
